# Construction and validation of a novel web-based nomogram for patients with lung cancer with bone metastasis: A real-world analysis based on the SEER database

**DOI:** 10.3389/fonc.2022.1075217

**Published:** 2022-12-09

**Authors:** Mengchen Yin, Sisi Guan, Xing Ding, Ruoyu Zhuang, Zhengwang Sun, Tao Wang, Jiale Zheng, Lin Li, Xin Gao, Haifeng Wei, Junming Ma, Quan Huang, Jianru Xiao, Wen Mo

**Affiliations:** ^1^ Longhua Hospital, Shanghai University of Traditional Chinese Medicine, Shanghai, China; ^2^ Changzheng Hospital, Second Affiliated Hospital of Naval Medical University, Shanghai, China; ^3^ Department of Musculoskeletal Surgery, Shanghai Cancer Center, Fudan University, Shanghai, China; ^4^ Department of Orthopaedics, The Second Hospital of Anhui Medical University, Anhui, China

**Keywords:** lung cancer, bone metastases, SEER, overall survival, cancer-specific survival

## Abstract

**Purpose:**

Patients with lung cancer with bone metastasis (LCBM) often have a very poor prognosis. The purpose of this study is to characterize the prevalence and associated factors and to develop a prognostic nomogram to predict the overall survival (OS) and cancer-specific survival (CSS) for patients with LCBM using multicenter population-based data.

**Methods:**

Patients with LCBM at the time of diagnosis were identified using the Surveillance, Epidemiology, and End Results (SEER) Program database of the National Cancer Institute (NCI) from 2010 to 2015. Multivariable and univariate logistic regression analyses were performed to identify factors associated with all-cause mortality and lung cancer (LC)–specific mortality. The performance of the nomograms was evaluated with the calibration curves, area under the curve (AUC), and decision curve analysis (DCA). Kaplan–Meier analysis and log-rank tests were used to estimate the survival times of patients with LCBM.

**Results:**

We finally identified 26,367 patients with LCBM who were selected for survival analysis. Multivariate analysis demonstrated age, sex, T stage, N stage, grade, histology, radiation therapy, chemotherapy, primary site, primary surgery, liver metastasis, and brain metastasis as independent predictors for LCBM. The AUC values of the nomogram for the OS prediction were 0.755, 0.746, and 0.775 in the training cohort; 0.757, 0.763, and 0.765 in the internal validation cohort; and 0.769, 0.781, and 0.867 in the external validation cohort. For CSS, the values were 0.753, 0.753, and 0.757 in the training cohort; 0.753, 0.753, and 0.757 in the internal validation cohort; and 0.767, 0.774, and 0.872 in the external validation cohort.

**Conclusions:**

Our study constructs a new prognostic model and clearly presents the clinicopathological features and survival analysis of patients with LCBM. The result indicated that the nomograms had favorable discrimination, good consistency, and clinical benefits in patients. In addition, our constructed nomogram prediction models may assist physicians in evaluating individualized prognosis and deciding on treatment for patients.

## Introduction

As the most lethal cancer worldwide, there are approximately 1.8 million new patients with lung cancer (LC) diagnosed each year (1, 2). Bone is the most common and the earliest site of metastases from LC, and 30%–40% of patients with LC already have bone metastases (BMs) upon initial diagnosis, which is usually associated with a poor prognosis (3–5). The therapy for patients with lung cancer with bone metastasis (LCBM) is diverse, including primary tumor resection, metastatic surgery, chemotherapy, and radiotherapy. However, in many cases, the survival of patients with LCBM could not be accurately assessed, and individualized therapeutic scheme could not be provided, which leads to additional distress and poorer prognosis of patients.

In LCBM, tumor cells release cytokines and chemical mediators to stimulate the periosteum and bone, combined with the mechanical stress caused by tumor tissue in the osteolytic lesions, causing serious bone pain. Moreover, it increases the risk of complications referred to as skeletal-related events (SREs), including pathologic fracture, spinal cord compression, and hypercalcemia of malignancy. The main therapeutic options for treating LCBM are chemotherapy and radiotherapy. The therapy for BM from solid tumors has been revolutionized over the last few decades. Since the 1990s, bisphosphonates were introduced to treat BM and became a mainstay of the management of BM. Until around the year 2000, the appearance of denosumab challenged this dominance. Based on existing studies, denosumab was found to be more effective in reducing SREs. However, aforementioned treatment development in BM was mainly dedicated to reducing SREs and bone pain, and the increase of overall survival (OS) was still limited. Data are still limited on the epidemiology, signatures, and prognostic factors of LCBM in general (6–8).

For data visualization, nomograms can increase the accuracy of prognostic prediction in cancer, using the tumor size. The chart integration predicts the probability of events. In previous studies, nomograms have demonstrated its superior predictive ability to the TNM staging system (9–12). As far as we know, the current study is the first that developed nomograms based on a large-size number of patients, which was verified by internal and external validation sets to guarantee the reliability of the results.

The Surveillance, Epidemiology, and End Results (SEER) Program’s registry is maintained by the National Cancer Institute (NCI), which collects a large number of cancer-related survival data from 1973 based on the US population. The cancer-related data in the SEER Program were obtained from 18 population-based registries, covering approximately 26% of the US population. Compared with any single institution, the SEER database encompasses much more population-level cancer data based on the largest sample size worldwide (13–16).

In this study, we analyzed the data extracted from the SEER database to identify risk factors associated with prognosis. Then, we subsequently created nomograms as a comprehensive prognostic assessment system. Both internal and external validation cohorts were employed to ensure the nomogram’s accuracy and reliability.

## Materials and methods

### Patient population

**Figure 1:**
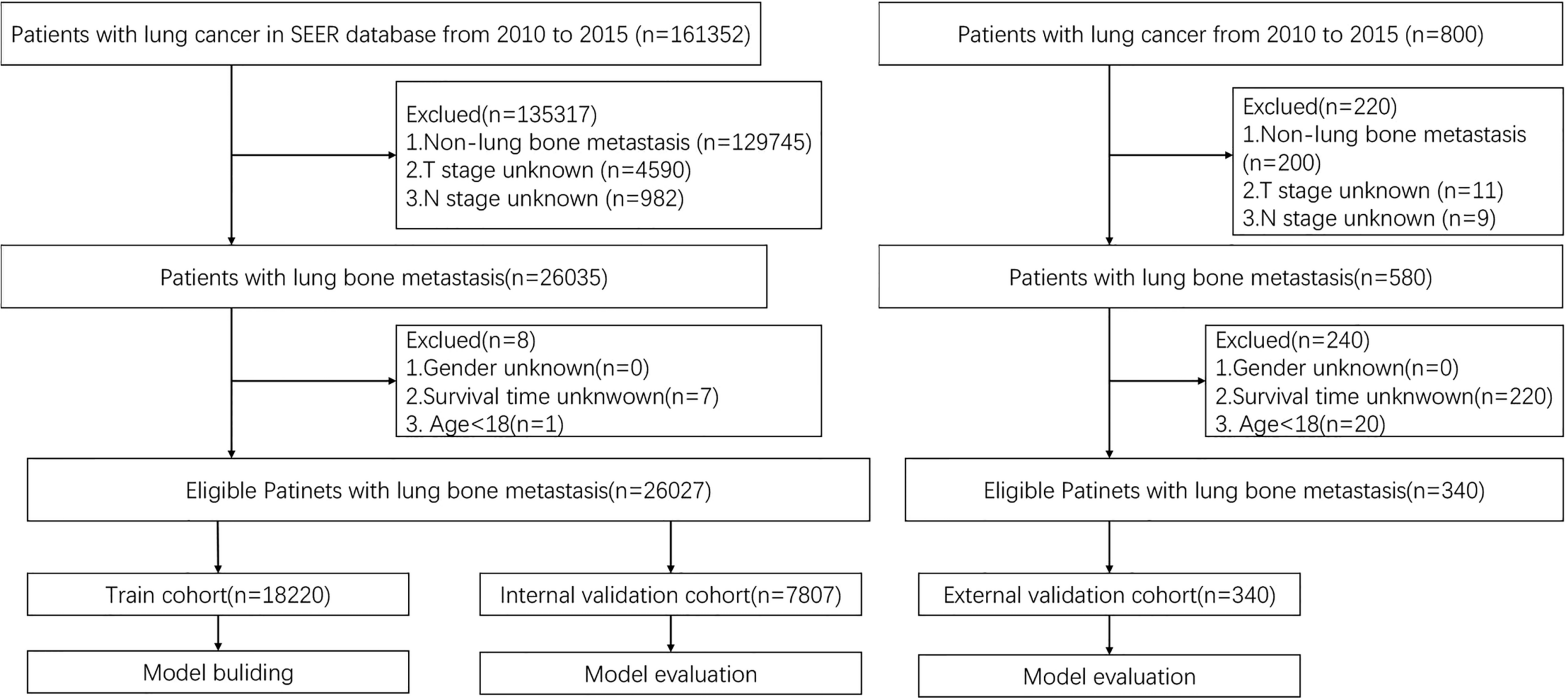
Flow chart of the study.

Patient data were extracted from the updated SEER database (https://seer.cancer.gov). We used the SEER Stat software version 8.3.9 published by SEER to identify eligible patients in this study. In addition, the data of eligible patients with LCBM in the external validation cohort treated in these institutions were acquired. The inclusion and exclusion criteria were the same as those for the training cohort from the SEER database. Eventually, we identified the prognostic factors of LCBM through the included patients. For each cohort, patients were randomly divided into the training set (70%) and the internal validation set (30%). Prognostic factors identified from patients in the training set were used to construct the nomogram that was validated by the internal validation set. The inclusion criteria are as follows: diagnosis with BM in 2010–2015 of all ages and patients with complete information recorded. To identify patients with metastatic LC to the bone, we selected cases with LCBM at the first diagnosis, for further research. In addition, patients who died during the study period were excluded. The independent external validation cohort was derived from patients with LCBM treated in four medical institutions (Longhua Hospital, Changzheng Hospital, Shanghai Cancer Center, and The Second Hospital of Anhui Medical University). The inclusion criteria are as follows: definite diagnosis of LCBM, 18 years of age and above, and complete follow-up information. **Figure 1** demonstrates the flowchart of the procedure. Analysis of the data from the SEER Program was exempted from medical ethics review, and no informed consent was required. The studies involving human participants were reviewed and approved by the Ethical Committee and Institutional Review Board. The patients/participants provided their written informed consent to participate in this study, and all procedures followed the Declaration of Helsinki.

### Data collection

The collected clinicopathological factors included the following: age, sex, histology/behavior, malignant, histrionic, radiation recode, chemotherapy recode, brain, liver, survival months, vital status recode, the SEER data on cause-specific death classification, the TNM stage, and pathological nodal grade. In accordance with the seventh edition of the American Joint Committee on Cancer staging system, we classified various clinicopathological factors, and the histological type of CRC patients was determined following the International Classification of Diseases for Oncology, third edition (ICD-O-3). The endpoints in our study were cancer-specific survival (CSS) and OS.

### Construction and validation of the prognostic scoring system

Univariable and multivariable Cox regression analyses were used to calculate the effects of variables on CSS and OS in the training, testing, and external validation cohorts. We formulated the nomogram based on the independent prognostic factors identified by the Cox multivariate analysis by employing R (version 4.0.1) with the rms package (available at http://CRAN.R-project.org/package=rms). The overall points for each patient in the training, testing, and external validation cohorts were calculated using the established nomogram, after which a Cox regression analysis of the entire cohort was carried out using the overall points as a parameter.

The Hosmer–Lemeshow test was used to evaluate the calibration of the nomogram and displayed in the form of the calibration curve. Both 1-, 3-, and 5-year CSS and OS can be estimated by the developed nomogram, respectively. Receiver operating characteristic (ROC) curve analyses were performed to test the performance evaluation of constructed nomograms by the areas under the ROC curves (AUCs), and Harrell’s concordance index (C-index) was applied to evaluate the predictive value of the constructed nomogram. At the same time, decision curve analysis (DCA) was applied to assess the nomogram in the current research that was a novel strategy for evaluating prognostic scoring system methods and has advantages over AUROC in clinical value evaluation.

### Statistical analysis

Statistical analysis was performed by SPSS v22.0 (IBM, Armonk, NY, USA) and R for Windows v4.0.5 (https://www.r-project.org). Chi-squared test was employed to analyze the categorical variables. The survival analysis was completed through the Kaplan–Meier method and log-rank test. The measure of the effect of each variable on CSS and OS is presented as the hazard ratio (HR) and was used to identify independent risk factors. HR greater than 1 indicated that the prognostic factor is unfavorable for survival, whereas HR smaller than 1 indicated that the prognostic factor is favorable for survival compared with the reference. A value of 1 revealed that there was no significant relationship between them. To minimize the influence of missing data, a backward stepwise method was used to further sort out prognostic factors selected in the multivariate Cox regression. The R statistical packages “rms”, “survival”, “Hmisc”, “MASS”, and “time ROC” were used to build a nomogram, plot calibration, and time-dependent ROC curves, whereas “rmda” was used to draw the DCA curves. All tests were two-sided, and P < 0.05 was considered statistically significant (17–19).

## Result

### Characteristics of the study population

A total of 26,027 patients with LCBM were included in our research. Meanwhile, 18,220 patients were enrolled into the training set, and the remaining 7,807 patients were enrolled into the internal validation set. The external validation set was composed by 340 eligible patients with LCBM. **Table 1** provides the characteristics of the 26,367 patients.

**Table 1:** Demographic and clinicopathological characteristics of patients in the training, internal validation, and external validation cohorts (N, %).

Variables	Level	SEER database						Patient database		
		Training set			Internal validation set			External validation set		
		Overall	Alive	Dead	Overall	Alive	Dead	Overall	Alive	Dead
		18,220	557	17,663	7,807	244	7,563	340	11	329
**Age (%)**	<65	7,857 (43.1)	338 (60.7)	7,519 (42.6)	3,394 (43.5)	144 (59.0)	3,250 (43.0)	136 (40.0)	7 (63.6)	129 (39.2)
	≥65	10,363 (56.9)	219 (39.3)	10,144 (57.4)	4,413 (56.5)	100 (41.0)	4313 (57.0)	204 (60.0)	4 (36.4)	200 (60.8)
**Sex (%)**	Male	10,479 (57.5)	266 (47.8)	10,213 (57.8)	4,483 (57.4)	126 (51.6)	4,357 (57.6)	201 (59.1)	5 (45.5)	196 (59.6)
	Female	7,741 (42.5)	291 (52.2)	7,450 (42.2)	3,324 (42.6)	118 (48.4)	3,206 (42.4)	139 (40.9)	6 (54.5)	133 (40.4)
**Histology (%)**	Squamous cell	2,617 (14.4)	47 (8.4)	2,570 (14.6)	1,165 (14.9)	23 (9.4)	1,142 (15.1)	53 (15.6)	2 (18.2)	51 (15.5)
	Adenocarcinoma	9,363 (51.4)	397 (71.3)	8,966 (50.8)	4,047 (51.8)	182 (74.6)	3,865 (51.1)	174 (51.2)	7 (63.6)	167 (50.8)
	Small cell	4,460 (24.5)	64 (11.5)	4,396 (24.9)	1,845 (23.6)	20 (8.2)	1,825 (24.1)	78 (22.9)	2 (18.2)	76 (23.1)
	Large cell	317 (1.7)	4 (0.7)	313 (1.8)	122 (1.6)	2 (0.8)	120 (1.6)	4 (1.2)	0 (0.0)	4 (1.2)
	Other	1,463 (8.0)	45 (8.1)	1,418 (8.0)	628 (8.0)	17 (7.0)	611 (8.1)	31 (9.1)	0 (0.0)	31 (9.4)
**T (%)**	T1	2,003 (11.0)	92 (16.5)	1,911 (10.8)	853 (10.9)	42 (17.2)	811 (10.7)	42 (12.4)	2 (18.2)	40 (12.2)
	T2	4,813 (26.4)	145 (26.0)	4,668 (26.4)	2,047 (26.2)	66 (27.0)	1,981 (26.2)	90 (26.5)	3 (27.3)	87 (26.4)
	T3	4,771 (26.2)	143 (25.7)	4,628 (26.2)	2,085 (26.7)	73 (29.9)	2,012 (26.6)	90 (26.5)	2 (18.2)	88 (26.7)
	T4	6,633 (36.4)	177 (31.8)	6,456 (36.6)	2,822 (36.1)	63 (25.8)	2,759 (36.5)	118 (34.7)	4 (36.4)	114 (34.7)
**N (%)**	N0	3,523 (19.3)	167 (30.0)	3,356 (19.0)	1,501 (19.2)	69 (28.3)	1,432 (18.9)	73 (21.5)	4 (36.4)	69 (21.0)
	N1	1,487 (8.2)	60 (10.8)	1,427 (8.1)	633 (8.1)	19 (7.8)	614 (8.1)	27 (7.9)	1 (9.1)	26 (7.9)
	N2	8,936 (49.0)	222 (39.9)	8,714 (49.3)	3,924 (50.3)	99 (40.6)	3,825 (50.6)	160 (47.1)	3 (27.3)	157 (47.7)
	N3	4,274 (23.5)	108 (19.4)	4,166 (23.6)	1,749 (22.4)	57 (23.4)	1,692 (22.4)	80 (23.5)	3 (27.3)	77 (23.4)
**M (%)**	M1a	465 (2.6)	16 (2.9)	449 (2.5)	216 (2.8)	10 (4.1)	206 (2.7)	7 (2.1)	0 (0.0)	7 (2.1)
	M1b	17,484 (96.0)	532 (95.5)	16,952 (96.0)	7,462 (95.6)	229 (93.9)	7,233 (95.6)	331 (97.4)	11 (100.0)	320 (97.3)
	M1NOS	271 (1.5)	9 (1.6)	262 (1.5)	129 (1.7)	5 (2.0)	124 (1.6)	2 (0.6)	0 (0.0)	2 (0.6)
**Primary site (%)**	Main bronchus	1,068 (5.9)	22 (3.9)	1,046 (5.9)	466 (6.0)	9 (3.7)	457 (6.0)	26 (7.6)	1 (9.1)	25 (7.6)
	Upper lobe	9,453 (51.9)	304 (54.6)	9,149 (51.8)	3,995 (51.2)	138 (56.6)	3,857 (51.0)	166 (48.8)	8 (72.7)	158 (48.0)
	Middle lobe	740 (4.1)	24 (4.3)	716 (4.1)	329 (4.2)	15 (6.1)	314 (4.2)	12 (3.5)	0 (0.0)	12 (3.6)
	Lower lobe	4,694 (25.8)	153 (27.5)	4,541 (25.7)	2,006 (25.7)	65 (26.6)	1,941 (25.7)	80 (23.5)	2 (18.2)	78 (23.7)
	Overlapping lesion of lung	187 (1.0)	5 (0.9)	182 (1.0)	91 (1.2)	2 (0.8)	89 (1.2)	7 (2.1)	0 (0.0)	7 (2.1)
	Lung, NOS	2,078 (11.4)	49 (8.8)	2,029 (11.5)	920 (11.8)	15 (6.1)	905 (12.0)	49 (14.4)	0 (0.0)	49 (14.9)
**Grade (%)**	I	291 (1.6)	16 (2.9)	275 (1.6)	144 (1.8)	6 (2.5)	138 (1.8)	7 (2.1)	0 (0.0)	7 (2.1)
	II	1,820 (10.0)	81 (14.5)	1,739 (9.8)	745 (9.5)	41 (16.8)	704 (9.3)	30 (8.8)	2 (18.2)	28 (8.5)
	III	4,346 (23.9)	140 (25.1)	4,206 (23.8)	1,876 (24.0)	54 (22.1)	1,822 (24.1)	82 (24.1)	1 (9.1)	81 (24.6)
	IV	608 (3.3)	6 (1.1)	602 (3.4)	262 (3.4)	3 (1.2)	259 (3.4)	12 (3.5)	0 (0.0)	12 (3.6)
	Unknown	11,155 (61.2)	314 (56.4)	10,841 (61.4)	4,780 (61.2)	140 (57.4)	4,640 (61.4)	209 (61.5)	8 (72.7)	201 (61.1)
**Primary surgery (%)**	Yes	331 (1.8)	37 (6.6)	294 (1.7)	125 (1.6)	21 (8.6)	104 (1.4)	9 (2.6)	3 (27.3)	6 (1.8)
	No	17,889 (98.2)	520 (93.4)	17,369 (98.3)	7,682 (98.4)	223 (91.4)	7,459 (98.6)	331 (97.4)	8 (72.7)	323 (98.2)
**Metastatic surgery (%)**	Yes	1,100 (6.0)	33 (5.9)	1,067 (6.0)	480 (6.1)	25 (10.2)	455 (6.0)	19 (5.6)	0 (0.0)	19 (5.8)
	No	17,120 (94.0)	524 (94.1)	16,596 (94.0)	7,327 (93.9)	219 (89.8)	7,108 (94.0)	321 (94.4)	11 (100.0)	310 (94.2)
**Radiation (%)**	Yes	9,653 (53.0)	296 (53.1)	9,357 (53.0)	4,095 (52.5)	137 (56.1)	3,958 (52.3)	170 (50.0)	2 (18.2)	168 (51.1)
	No	8,567 (47.0)	261 (46.9)	8,306 (47.0)	3,712 (47.5)	107 (43.9)	3,605 (47.7)	170 (50.0)	9 (81.8)	161 (48.9)
**Chemotherapy (%)**	Yes	10,778 (59.2)	450 (80.8)	10,328 (58.5)	4,618 (59.2)	203 (83.2)	4,415 (58.4)	189 (55.6)	10 (90.9)	179 (54.4)
	No	7,442 (40.8)	107 (19.2)	7,335 (41.5)	3,189 (40.8)	41 (16.8)	3,148 (41.6)	151 (44.4)	1 (9.1)	150 (45.6)
**Brain metastasis (%)**	Yes	4,380 (24.0)	116 (20.8)	4,264 (24.1)	1,813 (23.2)	58 (23.8)	1,755 (23.2)	90 (26.5)	1 (9.1)	89 (27.1)
	No	13,840 (76.0)	441 (79.2)	13,399 (75.9)	5,994 (76.8)	186 (76.2)	5,808 (76.8)	250 (73.5)	10 (90.9)	240 (72.9)
**Liver metastasis (%)**	Yes	5,649 (31.0)	83 (14.9)	5,566 (31.5)	2,473 (31.7)	33 (13.5)	2,440 (32.3)	105 (30.9)	3 (27.3)	102 (31.0)
	No	12,571 (69.0)	474 (85.1)	12,097 (68.5)	5,334 (68.3)	211 (86.5)	5,123 (67.7)	235 (69.1)	8 (72.7)	227 (69.0)

Most of the patients with LCBM from the SEER dataset were men (57.49%) and older than 65 years (56.77%). The most common histological type was adenocarcinoma (51.52%), and the most common primary site was the upper lobe (51.67%). Compared with living patients, those deceased were more likely to have had poor tumor histological type, poor tumor stage, poor tumor grade, and higher rates of liver metastasis. In terms of treatment, only 6.07% patients underwent metastatic surgeries, 59.15% patients have received chemotherapy, and 52.82% patients have received radiation therapy.

### Independent prognostic features in patients with lung cancer with bone metastasis

**Figure 2:**
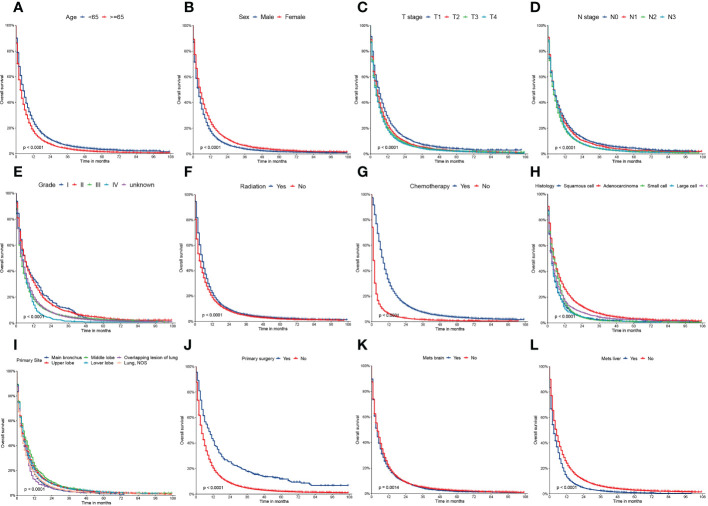
Kaplan–Meier curves of the overall survival (OS) factors: **(A)** age, **(B)** sex, **(C)** T stage, **(D)** N stage, **(E)** grade, **(F)** radiation, **(G)** chemotherapy, **(H)** histology, **(I)** primary site, **(J)** primary surgery, **(K)** mets-brain, and **(L)** mets-liver.

On univariable logistic regression analysis in the training cohort, there were 12 factors associated with OS that showed statistical significance (P < 0.05). These are age, sex, T stage, N stage, grade, radiation, chemotherapy, histology, primary site, primary surgery, brain metastasis (mets-brain), and liver metastasis (mets-liver). Then, the multivariate logistic regression analysis showed that age, sex, T stage, N stage, grade, radiation, chemotherapy, histology, primary site, primary surgery, mets-brain, and mets-liver were the independent predictors for the OS of patients with LCBM (**Tables 2** and **3**) **Figure 2** shows that all the above variables were significant in relation to OS.

**Table 2:** Univariate Cox regression model in the training, internal validation, and external validation cohorts of overall survival (OS) (N, %).

Variables	Level	SEER database				Patient database	
		Training cohort		Internal validation cohort		External validation cohort	
		HR (95% CI)	P	HR (95% CI)	P	HR (95% CI)	P
**Age**	<65						
	≥65	1.27 (1.23–1.31)	<0.001	1.3 (1.24–1.36)	<0.001	1.18 (0.95–1.48)	0.135
**Sex**	Male						
	Female	0.82 (0.79–0.84)	<0.001	0.84 (0.8–0.88)	<0.001	0.77 (0.61–0.96)	0.018
**T**	T1						
	T2	1.17 (1.11–1.24)	<0.001	1.18 (1.08–1.28)	<0.001	1.16 (0.8–1.69)	0.44
	T3	1.26 (1.2–1.33)	<0.001	1.32 (1.21–1.43)	<0.001	1.52 (1.04–2.21)	0.03
	T4	1.31 (1.24–1.38)	<0.001	1.33 (1.23–1.43)	<0.001	1.38 (0.96–1.98)	0.083
**N**	N0						
	N1	1.05 (0.99–1.12)	0.131	1.07 (0.97–1.17)	0.179	1.08 (0.69–1.7)	0.737
	N2	1.2 (1.16–1.25)	<0.001	1.16 (1.09–1.23)	0	1.2 (0.9–1.6)	0.207
	N3	1.15 (1.1–1.2)	<0.001	1.09 (1.01–1.17)	0.018	1.01 (0.73–1.4)	0.944
**M**	M1a						
	M1b	1.03 (0.94–1.13)	0.576	1.08 (0.94–1.24)	0.267	0.71 (0.34–1.51)	0.379
	M1NOS	0.99 (0.85–1.15)	0.861	0.95 (0.76–1.19)	0.65	0.69 (0.14–3.33)	0.644
**Grade**	I						
	II	1.04 (0.92–1.18)	0.534	1.12 (0.93–1.34)	0.232	1.19 (0.52–2.73)	0.68
	III	1.4 (1.24–1.59)	<0.001	1.5 (1.27–1.79)	<0.001	1.88 (0.86–4.07)	0.112
	IV	1.55 (1.35–1.79)	<0.001	1.59 (1.29–1.95)	<0.001	2.71 (1.06–6.93)	0.037
	Unknown	1.35 (1.2–1.52)	<0.001	1.4 (1.18–1.66)	<0.001	1.64 (0.77–3.49)	0.2
**Radiation**	Yes						
	No	1.21 (1.18–1.25)	<0.001	1.2 (1.15–1.26)	<0.001	1.1 (0.88–1.37)	0.394
**Chemotherapy**	Yes						
	No	3.04 (2.95–3.14)	<0.001	3.19 (3.04–3.35)	<0.001	3.23 (2.57–4.06)	<0.001
**Histology**	Squamous cell						
	Adenocarcinoma	0.69 (0.66–0.72)	<0.001	0.65 (0.61–0.69)	<0.001	0.74 (0.54–1.01)	0.06
	Small cell	1.03 (0.91–1.16)	0.637	1.07 (0.89–1.3)	0.456	1.64 (0.59–4.55)	0.343
	Large cell	0.88 (0.83–0.94)	<0.001	0.89 (0.81–0.98)	0.021	1.31 (0.83–2.05)	0.246
	Other	0.89 (0.85–0.93)	<0.001	0.85 (0.79–0.92)	<0.001	0.99 (0.69–1.42)	0.953
**Primary site**	Main bronchus						
	Upper lobe	0.85 (0.8–0.91)	<0.001	0.81 (0.73–0.89)	<0.001	0.63 (0.41–0.97)	0.037
	Middle lobe	0.8 (0.73–0.88)	<0.001	0.78 (0.67–0.9)	0.001	0.78 (0.39–1.56)	0.484
	Lower lobe	0.87 (0.81–0.93)	<0.001	0.78 (0.71–0.87)	<0.001	0.69 (0.44–1.08)	0.107
	Overlapping lesion of lung	1.03 (0.88–1.21)	0.714	0.82 (0.65–1.02)	0.079	0.81 (0.35–1.89)	0.631
	Lung, NOS	1.01 (0.94–1.09)	0.7	1.05 (0.94–1.17)	0.401	1.02 (0.63–1.66)	0.922
**Primary surgery**	Yes						
	No	1.79 (1.59–2.01)	<0.001	1.85 (1.52–2.25)	<0.001	3.17 (1.41–7.13)	0.005
**Metastatic surgery**	Yes						
	No	1.07 (1–1.13)	0.041	1.2 (1.09–1.32)	<0.001	0.87 (0.55–1.39)	0.564
**Brain metastasis**	Yes						
	No	0.94 (0.91–0.98)	0.001	0.94 (0.89–0.99)	0.014	0.73 (0.57–0.93)	0.011
**Liver metastasis**	Yes						
	No	0.73 (0.71–0.75)	<0.001	0.7 (0.66–0.73)	<0.001	0.73 (0.58–0.93)	0.009

HR, hazard ratio.

**Table 3:** Multivariate Cox regression model in the training, internal validation, and external validation cohorts of overall survival (OS) (N, %).

Variables	Levels	SEER database				Patient database	
		Training cohort		Internal validation cohort		External validation cohort	
		HR (95% CI)	P	HR (95% CI)	P	HR (95% CI)	P
**Age**	<65						
	≥65	1.18 (1.14–1.21)	<0.001	1.15 (1.1–1.21)	<0.001	NA	NA
**Sex**	Male						
	Female	0.84 (0.81–0.86)	<0.001	0.87 (0.83–0.91)	<0.001	0.75 (0.59–0.94)	0.0118
**T**	T1						
	T2	1.13 (1.07–1.19)	<0.001	1.15 (1.05–1.24)	0.0013	1.05 (0.72–1.56)	0.7873
	T3	1.21 (1.15–1.28)	<0.001	1.24 (1.15–1.35)	<0.001	1.27 (0.86–1.88)	0.2215
	T4	1.23 (1.17–1.3)	<0.001	1.22 (1.12–1.32)	<0.001	1.07 (0.73–1.57)	0.7204
**N**	N0						
	N1	1.15 (1.08–1.22)	<0.001	1.04 (0.95–1.15)	0.3712	NA	NA
	N2	1.26 (1.21–1.31)	<0.001	1.25 (1.17–1.33)	<0.001	NA	NA
	N3	1.28 (1.22–1.34)	<0.001	1.21 (1.13–1.3)	<0.001	NA	NA
**M**	M1a						
	M1b	NA	NA	NA	NA	NA	NA
	M1NOS	NA	NA	NA	NA	NA	NA
**Grade**	I						
	II	1.06 (0.94–1.21)	0.3446	1.2 (1–1.44)	0.0526	2.55 (1.08–6.04)	0.0333
	III	1.35 (1.19–1.52)	<0.001	1.51 (1.27–1.79)	<0.001	3.13 (1.39–7.05)	0.006
	IV	1.38 (1.19–1.59)	<0.001	1.55 (1.25–1.91)	<0.001	4.04 (1.49–10.99)	0.0062
	Unknown	1.29 (1.15–1.46)	<0.001	1.43 (1.2–1.69)	<0.001	2.71 (1.22–5.99)	0.0139
**Radiation**	Yes						
	No	1.12 (1.08–1.15)	<0.001	1.09 (1.04–1.14)	<0.001	NA	NA
**Chemotherapy**	Yes						
	No	3.24 (3.13–3.34)	<0.001	3.37 (3.2–3.54)	<0.001	3.97 (3.09–5.11)	<0.001
**Histology**	Squamous cell						
	Adenocarcinoma	0.78 (0.74–0.81)	<0.001	0.78 (0.73–0.83)	<0.001	NA	NA
	Small cell	1.05 (0.94–1.19)	0.3786	1.13 (0.94–1.37)	0.1938	NA	NA
	Large cell	0.89 (0.83–0.95)	<0.001	0.89 (0.81–0.99)	0.0286	NA	NA
	Other	0.97 (0.92–1.02)	0.299	0.95 (0.88–1.03)	0.2403	NA	NA
**Primary site**	Main bronchus						
	Upper lobe	0.89 (0.83–0.95)	<0.001	0.9 (0.82–0.99)	0.0359	0.63 (0.4–0.97)	0.038
	Middle lobe	0.79 (0.71–0.87)	<0.001	0.84 (0.73–0.97)	0.02	0.76 (0.37–1.55)	0.4503
	Lower lobe	0.9 (0.84–0.96)	0.0027	0.87 (0.78–0.96)	0.0067	0.6 (0.37–0.97)	0.0373
	Overlapping lesion of lung	1.04 (0.89–1.21)	0.6493	0.84 (0.67–1.05)	0.1334	0.79 (0.33–1.88)	0.6003
	Lung, NOS	0.98 (0.91–1.06)	0.6851	1.13 (1.01–1.27)	0.034	0.97 (0.59–1.6)	0.9153
**Primary surgery**	Yes						
	No	1.72 (1.53–1.94)	<0.001	1.79 (1.47–2.18)	<0.001	2.25 (0.98–5.17)	0.0562
**Metastatic surgery**	Yes						
	No	1.06 (0.99–1.13)	0.0728	1.07 (0.98–1.18)	0.1481	NA	NA
**Brain metastasis**	Yes						
	No	0.85 (0.82–0.88)	<0.001	0.83 (0.79–0.88)	<0.001	0.78 (0.61–1.01)	0.0621
**Liver metastasis**	Yes						
	No	0.74 (0.72–0.77)	<0.001	0.71 (0.67–0.74)	<0.001	0.67 (0.52–0.86)	0.0022

N/A, Not Applicable.

**Figure 3:**
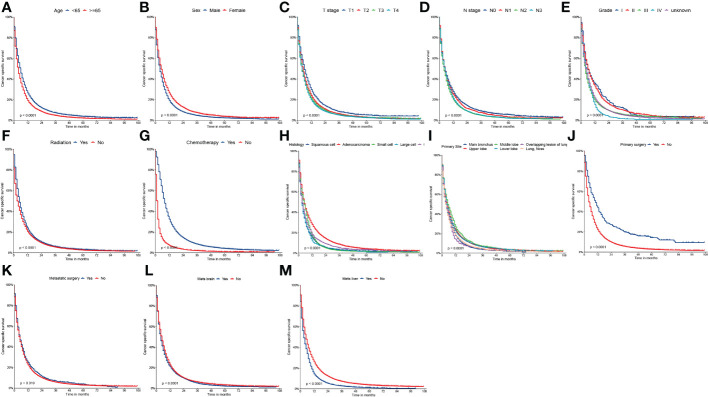
Kaplan-Meier curves of CSS **(A)** age, **(B)** sex, **(C)** T stage, **(D)** N stage, **(E)** grade, **(F)** radiation, **(G)** chemotherapy, **(H)** histology, **(I)** primary site, **(J)** primary surgery, **(K)** metastatic surgery, **(L)** mets-brain and **(M)** mets-liver.

On univariable logistic regression analysis in the training cohort, there were 13 factors associated with CSS that showed statistical significance (P < 0.05). These were age, sex, T stage, N stage, grade, radiation, chemotherapy, histology, primary site, primary surgery, metastatic surgery, mets-brain, and mets-liver. Then, the multivariate logistic regression analysis showed that age, sex, T stage, N stage, grade, radiation, chemotherapy, histology, primary site, primary surgery, metastatic surgery, mets-brain, and mets-liver were the independent predictors predicting the CSS for patients with LCBM (**Tables 4** and **5**). **Figure 3** shows that all the above variables were significant in relation to CSS.

**Table 4:** Univariate Cox regression model in the training, internal validation, and external validation cohorts of cancer-specific survival (CSS) (N, %).

Variables	Level	SEER database				Patient database	
		Training cohort		Internal validation cohort		External validation cohort	
		HR (95% CI)	P	HR (95% CI)	P	HR (95% CI)	P
**Age**	<65						
	≥65	1.25 (1.21–1.29)	<0.001	1.28 (1.22–1.34)	<0.001	1.11 (0.89–1.4)	0.36
**Sex**	Male						
	Female	0.82 (0.8–0.85)	<0.001	0.85 (0.81–0.89)	<0.001	0.72 (0.58–0.91)	0.006
**T**	T1						
	T2	1.18 (1.12–1.24)	<0.001	1.2 (1.11–1.31)	<0.001	1.11 (0.76–1.64)	0.58
	T3	1.27 (1.2–1.34)	<0.001	1.32 (1.22–1.44)	<0.001	1.43 (0.98–2.11)	0.066
	T4	1.32 (1.25–1.39)	<0.001	1.36 (1.25–1.47)	<0.001	1.31 (0.91–1.9)	0.147
**N**	N0						
	N1	1.06 (0.99–1.13)	0.092	1.08 (0.98–1.19)	0.139	1.07 (0.67–1.69)	0.783
	N2	1.21 (1.17–1.27)	<0.001	1.17 (1.1–1.25)	<0.001	1.13 (0.84–1.52)	0.406
	N3	1.16 (1.1–1.21)	<0.001	1.11 (1.03–1.19)	0.006	0.98 (0.7–1.37)	0.911
**M**	M1a						
	M1b	1.07 (0.97–1.17)	0.204	1.09 (0.95–1.26)	0.229	0.67 (0.32–1.43)	0.301
	M1NOS	1.02 (0.87–1.19)	0.808	0.93 (0.74–1.17)	0.517	0.7 (0.14–3.35)	0.65
**Grade**	I						
	II	1.05 (0.92–1.2)	0.471	1.14 (0.94–1.37)	0.179	1.6 (0.62–4.17)	0.332
	III	1.42 (1.25–1.61)	<0.001	1.54 (1.29–1.84)	<0.001	2.58 (1.04–6.39)	0.04
	IV	1.57 (1.36–1.82)	<0.001	1.64 (1.33–2.03)	<0.001	3.77 (1.32–10.76)	0.013
	Unknown	1.37 (1.21–1.55)	<0.001	1.41 (1.19–1.69)	<0.001	2.1 (0.86–5.13)	0.101
**Radiation**	Yes						
	No	1.19 (1.16–1.23)	<0.001	1.17 (1.12–1.23)	<0.001	1.08 (0.86–1.34)	0.525
**Chemotherapy**	Yes						
	No	3.02 (2.92–3.12)	<0.001	3.15 (3–3.31)	<0.001	3.19 (2.52–4.04)	<0.001
**Histology**	Squamous cell						
	Adenocarcinoma	0.69 (0.66–0.73)	<0.001	0.64 (0.6–0.69)	<0.001	0.77 (0.55–1.07)	0.114
	Small cell	1.04 (0.92–1.17)	0.515	1.08 (0.89–1.31)	0.425	1.78 (0.64–4.97)	0.268
	Large cell	0.9 (0.84–0.96)	0.001	0.88 (0.8–0.98)	0.016	1.29 (0.8–2.07)	0.296
	Other	0.9 (0.85–0.94)	<0.001	0.85 (0.79–0.92)	<0.001	1.04 (0.72–1.51)	0.83
**Primary site**	Main bronchus						
	Upper lobe	0.84 (0.79–0.9)	<0.001	0.82 (0.74–0.9)	<0.001	0.6 (0.39–0.92)	0.019
	Middle lobe	0.78 (0.71–0.86)	<0.001	0.79 (0.68–0.92)	0.002	0.71 (0.35–1.45)	0.352
	Lower lobe	0.85 (0.79–0.91)	<0.001	0.79 (0.71–0.87)	<0.001	0.66 (0.42–1.04)	0.072
	Overlapping lesion of lung	1.01 (0.86–1.19)	0.895	0.87 (0.69–1.09)	0.23	0.82 (0.35–1.9)	0.641
	Lung, NOS	1 (0.93–1.08)	0.904	1.05 (0.94–1.18)	0.392	0.94 (0.57–1.53)	0.797
**Primary surgery**	Yes						
	No	1.82 (1.61–2.05)	<0.001	1.9 (1.55–2.33)	<0.001	2.96 (1.31–6.66)	0.009
**Metastatic surgery**	Yes						
	No	1.08 (1.01–1.15)	0.018	1.19 (1.08–1.31)	<0.001	0.81 (0.51–1.3)	0.385
**Brain metastasis**	Yes						
	No	0.93 (0.9–0.96)	<0.001	0.92 (0.87–0.97)	0.003	0.7 (0.55–0.9)	0.006
**Liver metastasis**	Yes						
	No	0.72 (0.7–0.74)	<0.001	0.69 (0.65–0.72)	<0.001	0.7 (0.55–0.89)	0.004

**Table 5:** Multivariate Cox regression model in the training, internal validation, and external validation cohorts of cancer-specific survival (CSS) (N, %).

Variables	Levels	SEER database				Patient database	
		Training cohort		Internal validation cohort		External validation cohort	
		HR (95% CI)	P	HR (95% CI)	P	HR (95% CI)	P
**Age**	<65						
	≥65	1.16 (1.13–1.2)	<0.001	1.14 (1.08–1.19)	<0.001	NA	NA
**Sex**	Male						
	Female	0.84 (0.82–0.87)	<0.001	0.88 (0.84–0.92)	<0.001	0.69 (0.54–0.87)	0.0019
**T**	T1						
	T2	1.14 (1.07–1.2)	<0.001	1.17 (1.07–1.27)	<0.001	NA	NA
	T3	1.21 (1.15–1.28)	<0.001	1.25 (1.14–1.36)	<0.001	NA	NA
	T4	1.24 (1.17–1.31)	<0.001	1.24 (1.14–1.35)	<0.001	NA	NA
**N**	N0						
	N1	1.15 (1.08–1.23)	<0.001	1.05 (0.95–1.16)	0.309	NA	NA
	N2	1.26 (1.21–1.32)	<0.001	1.25 (1.17–1.33)	<0.001	NA	NA
	N3	1.28 (1.22–1.35)	<0.001	1.22 (1.14–1.32)	<0.001	NA	NA
**M**	M1a						
	M1b	NA	NA	NA	NA	NA	NA
	M1NOS	NA	NA	NA	NA	NA	NA
**Grade**	I						
	II	1.07 (0.94–1.22)	0.2936	1.21 (1–1.47)	0.0449	3.48 (1.3–9.29)	0.0128
	III	1.36 (1.2–1.54)	<0.001	1.53 (1.27–1.83)	<0.001	4.41 (1.74–11.19)	0.0018
	IV	1.39 (1.2–1.62)	<0.001	1.57 (1.26–1.96)	<0.001	5.68 (1.89–17.08)	0.002
	Unknown	1.3 (1.15–1.48)	<0.001	1.42 (1.19–1.7)	<0.001	3.57 (1.42–8.96)	0.0068
**Radiation**	Yes						
	No	1.1 (1.06–1.13)	<0.001	1.07 (1.02–1.12)	0.0108	NA	NA
**Chemotherapy**	Yes						
	No	3.23 (3.12–3.34)	<0.001	3.35 (3.18–3.52)	<0.001	4.06 (3.13–5.27)	<0.001
**Histology**	Squamous cell						
	Adenocarcinoma	0.79 (0.75–0.82)	<0.001	0.77 (0.72–0.83)	<0.001	NA	NA
	Small cell	1.06 (0.94–1.2)	0.315	1.13 (0.93–1.37)	0.2294	NA	NA
	Large cell	0.9 (0.84–0.97)	0.0031	0.89 (0.8–0.98)	0.0226	NA	NA
	Other	0.98 (0.93–1.03)	0.4556	0.95 (0.88–1.03)	0.221	NA	NA
**Primary site**	Main bronchus						
	Upper lobe	0.88 (0.82–0.94)	<0.001	0.92 (0.83–1.01)	0.0901	0.6 (0.39–0.94)	0.025
	Middle lobe	0.77 (0.7–0.85)	<0.001	0.87 (0.75–1)	0.0567	0.71 (0.34–1.48)	0.36
	Lower lobe	0.89 (0.83–0.95)	<0.001	0.88 (0.79–0.98)	0.0162	0.59 (0.37–0.96)	0.0329
	Overlapping lesion of lung	1.02 (0.87–1.2)	0.8241	0.9 (0.72–1.14)	0.3853	0.86 (0.36–2.04)	0.7357
	Lung, NOS	0.98 (0.9–1.05)	0.5287	1.14 (1.02–1.28)	0.0262	0.93 (0.56–1.54)	0.7666
**Primary surgery**	Yes						
	No	1.74 (1.55–1.97)	<0.001	1.86 (1.51–2.28)	<0.001	2.1 (0.91–4.81)	0.0802
**Metastatic surgery**	Yes						
	No	1.08 (1.01–1.15)	0.0255	1.07 (0.97–1.18)	0.1971	NA	NA
**Brain metastasis**	Yes						
	No	0.85 (0.82–0.88)	<0.001	0.83 (0.78–0.88)	<0.001	0.75 (0.57–0.97)	0.0274
**Liver metastasis**	Yes						
	No	0.73 (0.71–0.76)	<0.001	0.7 (0.66–0.73)	<0.001	0.65 (0.5–0.85)	0.0013

N/A, Not Applicable.

### Construction of the nomogram

**Figure 4:**
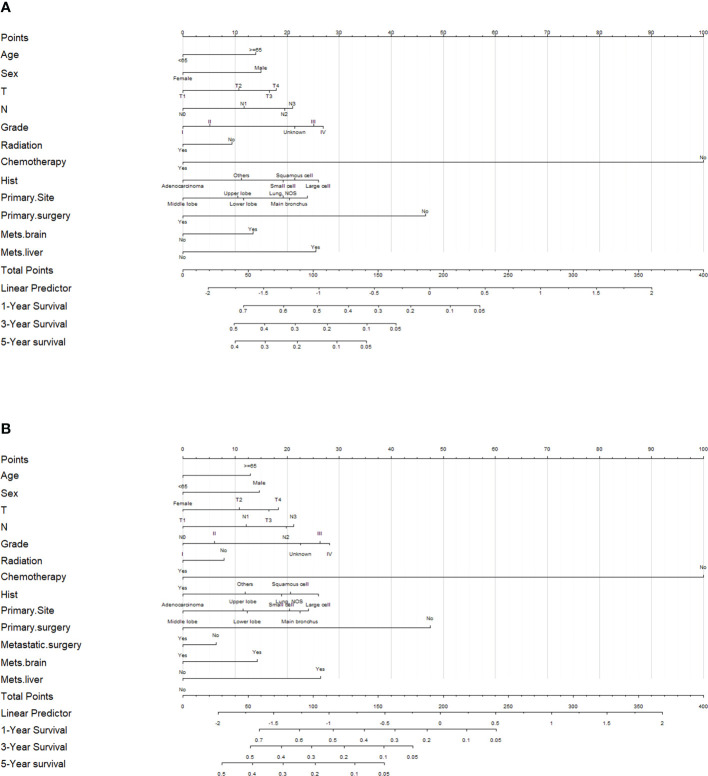
Evaluation of the overall survival (OS)- and cancer-specific survival (CSS)-associated nomograms for patients with lung cancer with bone metastasis (LCBM). **(A)** OS nomogram integrating age, sex, T stage, N stage, grade, radiation, chemotherapy, histology, primary site, primary surgery, mets-brain, and mets-liver for predicting 1-, 3-, and 5-year OS rates. **(B)** CSS nomogram integrating age, sex, T stage, N stage, grade, radiation, chemotherapy, histology, primary site, primary surgery, metastatic surgery, mets-brain, and mets-liver for predicting 1-, 3-, and 5-year CSS rates.

To predict 1-, 3-, and 5-year OS and CSS of patients with LCBM, we built a nomogram in accordance with the major prognostic factors identified by the multivariable Cox regression analysis. Total points can be calculated by adding up the values for each variable corresponding to nomogram points. Subsequently, the value of total points corresponds vertically to survival chances at multiple time points (**Figure 4**).

### Comparison of the values of area under the curves of the nomogram with TNM stage

**Figure 5:**
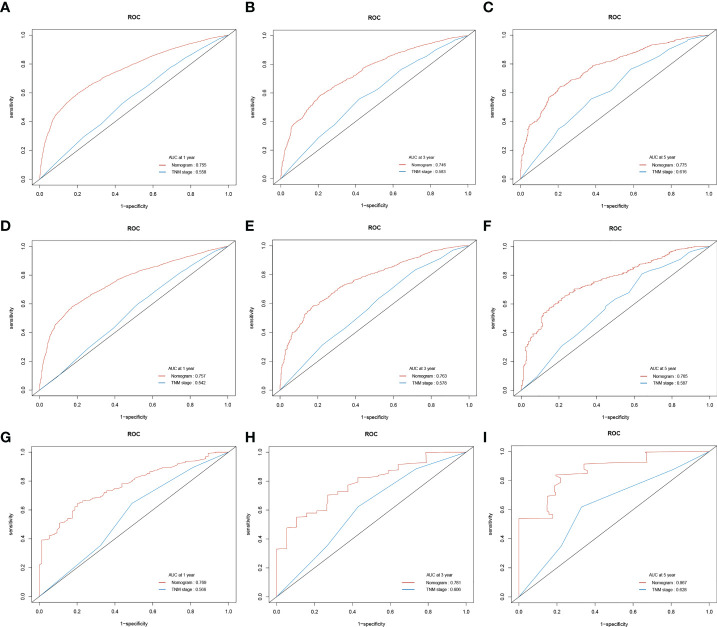
The receiver operating characteristic (ROC) curve of the TNM stage and the overall survival (OS) nomogram. **(A–C)** The area under the curve (AUC) values of ROC predicted 1-, 3-, and 5-year OS rates of the nomogram and TNM stage in the training cohort. **(D–F)** AUC values of ROC predicted 1-, 3-, and 5-year OS rates of the nomogram and TNM stage in the internal validation cohort. **(G–I)** AUC values of ROC predicted 1-, 3-, and 5-year OS rates of the nomogram and TNM stage in the external validation cohort.

We conducted the time-dependent ROC analyses at 1, 3, and 5 years in the training, internal validation, and external validation cohorts. In the training cohort, the AUC values of the nomogram for the prediction of OS (AUCOS) were 0.755, 0.746, and 0.775, compared with 0.558, 0.583, and 0.616 for the AUC values of TNM stage (AUCTNM), respectively. In the internal validation cohort, AUCOS were 0.757, 0.763, and 0.765, compared with 0.542, 0.578, and 0.587 for the AUCTNM, respectively. In the external validation cohort, AUCOS were 0.769, 0.781, and 0.867, compared with 0.566, 0.606, and 0.628 for the AUCTNM, respectively (**Figure 5**).

**Figure 6:**
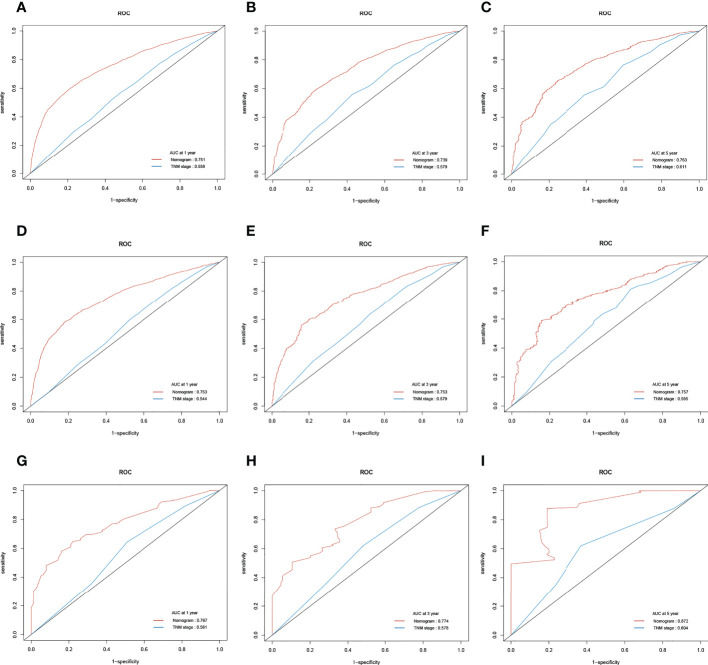
The receiver operating characteristic (ROC) curve of the TNM stage and the cancer-specific survival (CSS) nomogram. **(A–C)** The area under the curve (AUC) values of ROC predicted 1-, 3-, and 5-year CSS rates of the nomogram and TNM stage in the training cohort. **(D–F)** AUC values of ROC predicted 1-, 3-, and 5-year CSS rates of the nomogram and TNM stage in the internal validation cohort. **(G–I)** AUC values of ROC predicted 1-, 3-, and 5-year CSS rates of the nomogram and TNM stage in the external validation cohort.

Likewise, in the training cohort, the AUC values of the nomogram for the prediction of CSS (AUCCSS) were 0.753, 0.753, and 0.757, compared with 0.558, 0.579, and 0.611 for the AUCTNM stage, respectively. In the internal validation cohort, AUCCSS were 0.753, 0.753, and 0.757, compared with 0.544, 0.579, and 0.595 for the AUCTNM, respectively. In the external validation cohort, AUCCSS were 0.767, 0.774, and 0.872, compared with 0.561, 0.578, and 0.604 for the AUCTNM, respectively (**Figure 6**). The results showed that the novel prognostic scoring system had better efficacy in predicting the prognosis of patients with LCBM than TNM stage.

### Evaluation and validation of the overall survival and cancer-specific survival prediction nomograms using receiver operating characteristic curves

We also used time-dependent ROC curves and the AUC to validate the discrimination ability of nomograms. In the training cohort, the 1-, 3-, and 5-year AUC values of nomograms predicting OS were 0.755, 0.746, and 0.775, respectively. In the internal validation cohort, the AUC values for OS were 0.757, 0.763, and 0.765, respectively. In the external validation cohort, the AUC values for OS were 0.769, 0.781, and 0.867, respectively.

**Figure 7:**
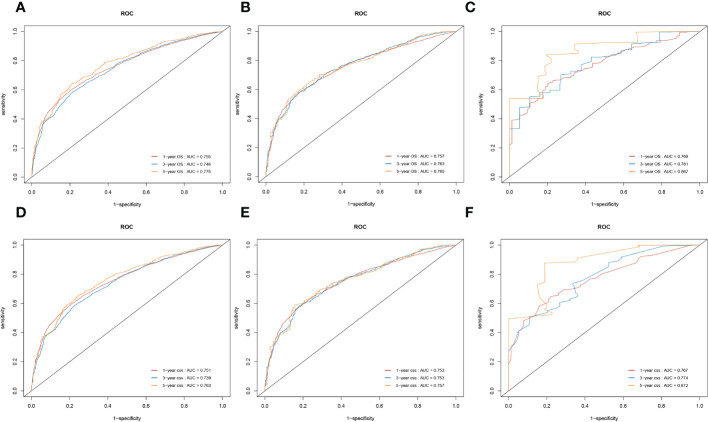
Nomograms of time-dependent receiver operating characteristic (ROC) curves for the overall survival (OS) and cancer-specific survival (CSS) prediction of patients with lung cancer with bone metastasis (LCBM). **(A–C)** The training, internal validation, and external validation cohorts for the OS. **(D–F)** The training, internal validation, and external validation cohorts for the CSS.

Likewise, in the training cohorts, the 1-, 3-, and 5-year AUC values of nomograms predicting for CSS were 0.751, 0.739, and 0.763, respectively. In the internal validation cohorts, the AUC values for CSS were 0.753, 0.753, and 0.757, respectively. In the external validation cohorts, the AUC values for CSS were 0.767, 0.774, and 0.872, respectively. The results showed that the novel prognostic scoring system had a favorable predictive sensitivity (**Figure 7**).

C-index values for the prediction of OS and CSS were also used to evaluate the discriminatory power of the nomogram. The C-index values for OS were 0.717 (95% CI, 0.715–0.719), 0.721 (95% CI, 0.718–0.725), and 0.731 (95% CI, 0.716–0.746) in the training, internal validation, and external validation cohorts, respectively. Likewise, the C-index values for CSS were 0.716 (95% CI, 0.714–0.718), 0.720 (95% CI, 0.716–0.723), and 0.731 (95% CI, 0.715–0.746) in the training, internal validation, and external validation cohorts. The result indicated that the nomogram had favorable discrimination in patients with LCBM.

**Figure 8:**
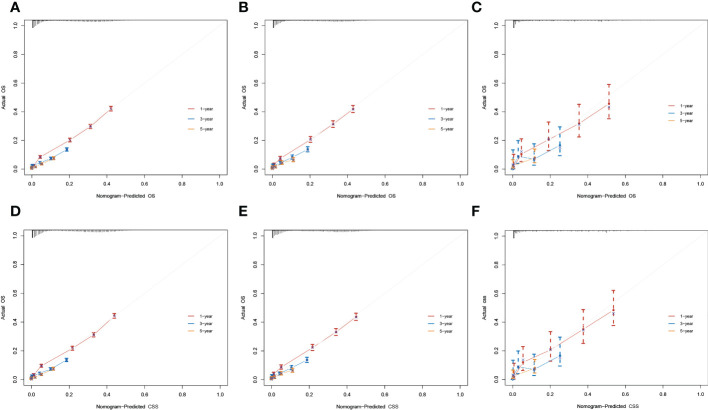
Calibration curves for 1-, 3-, and 5-year overall survival (OS) and cancer-specific survival (CSS) rates of the nomogram predictions. **(A–C)** The training, internal validation, and external validation cohorts for the OS. **(D–F)** The training, internal validation, and external validation cohorts for the CSS.

**Figure 9:**
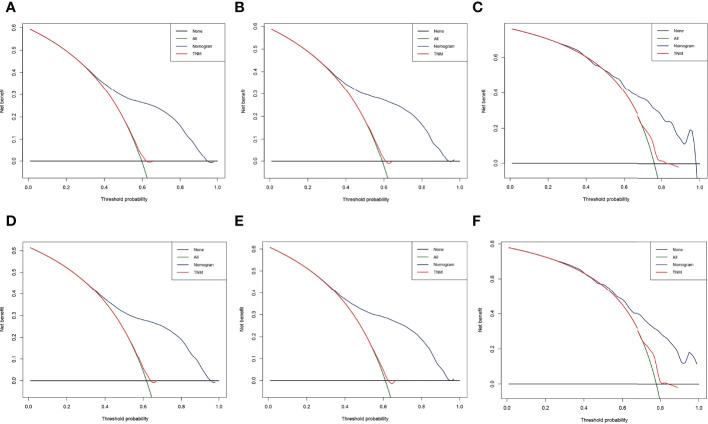
Decision curve analysis (DCA) of the nomogram and TNM stage for the overall survival (OS) and cancer-specific survival (CSS) prediction of patients with lung cancer with bone metastasis (LCBM). **(A)** The training, **(B)** internal validation, and **(C)** external validation cohorts for the OS. **(D)** The training, **(E)** internal validation, and **(F)** external validation cohorts for the CSS.

The result of calibration curves for the nomogram showed no obvious deviations from the reference line, indicating a high degree of credibility (**Figure 8**). In addition, DCA of the nomogram and TNM stage for the OS and CSS prediction of patients was used to evaluate the clinical value. The result of DCA indicated that the nomogram had better clinical outcome values compared with the TNM staging system with higher net benefits (**Figure 9**).

### Web-based nomogram

**Figure 10:**
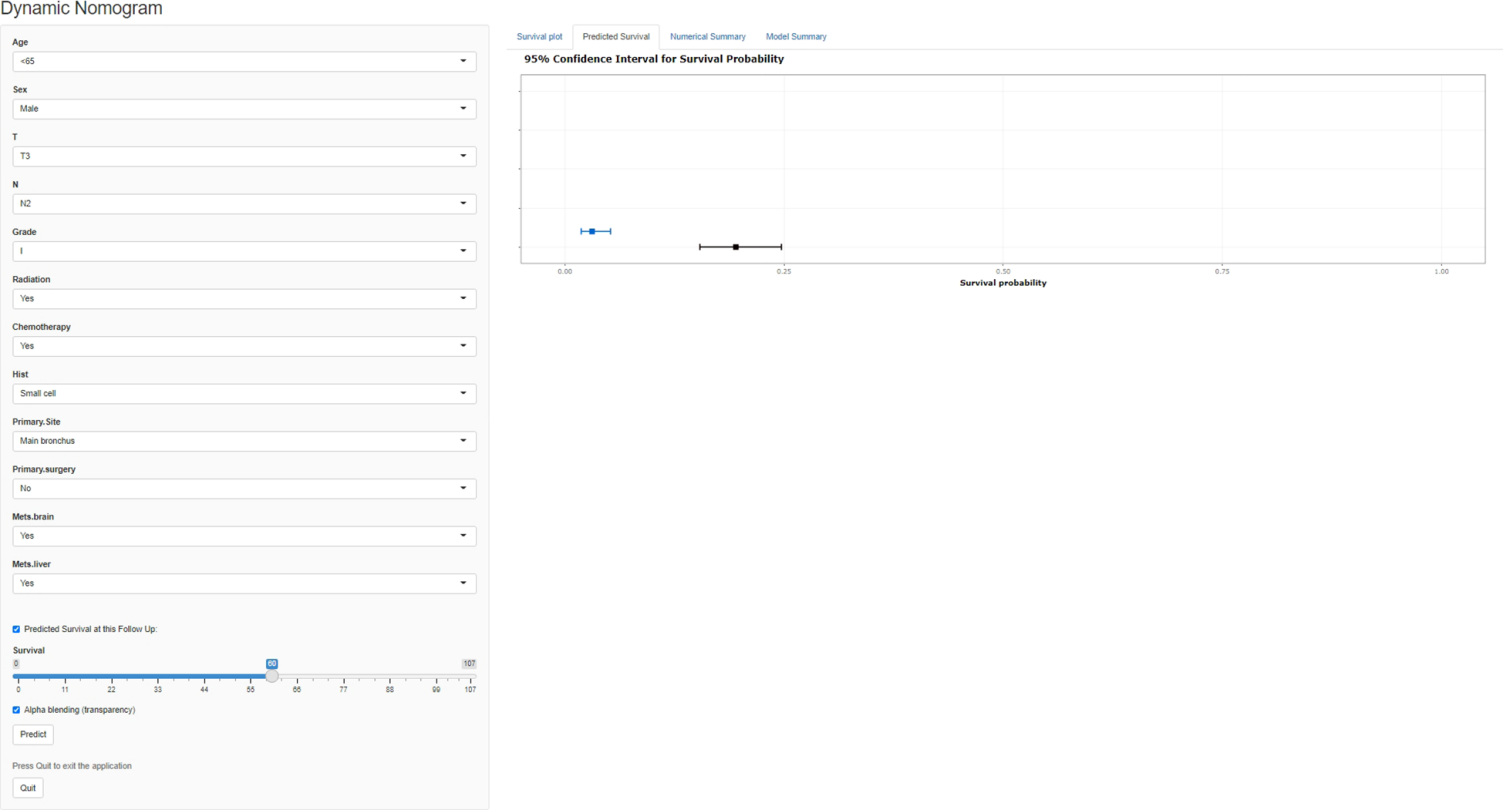
A web-based nomogram for predicting postoperative overall survival (OS). The line segments of the graphical summary show the approximate range of overall survival rates.

**Figure 11:**
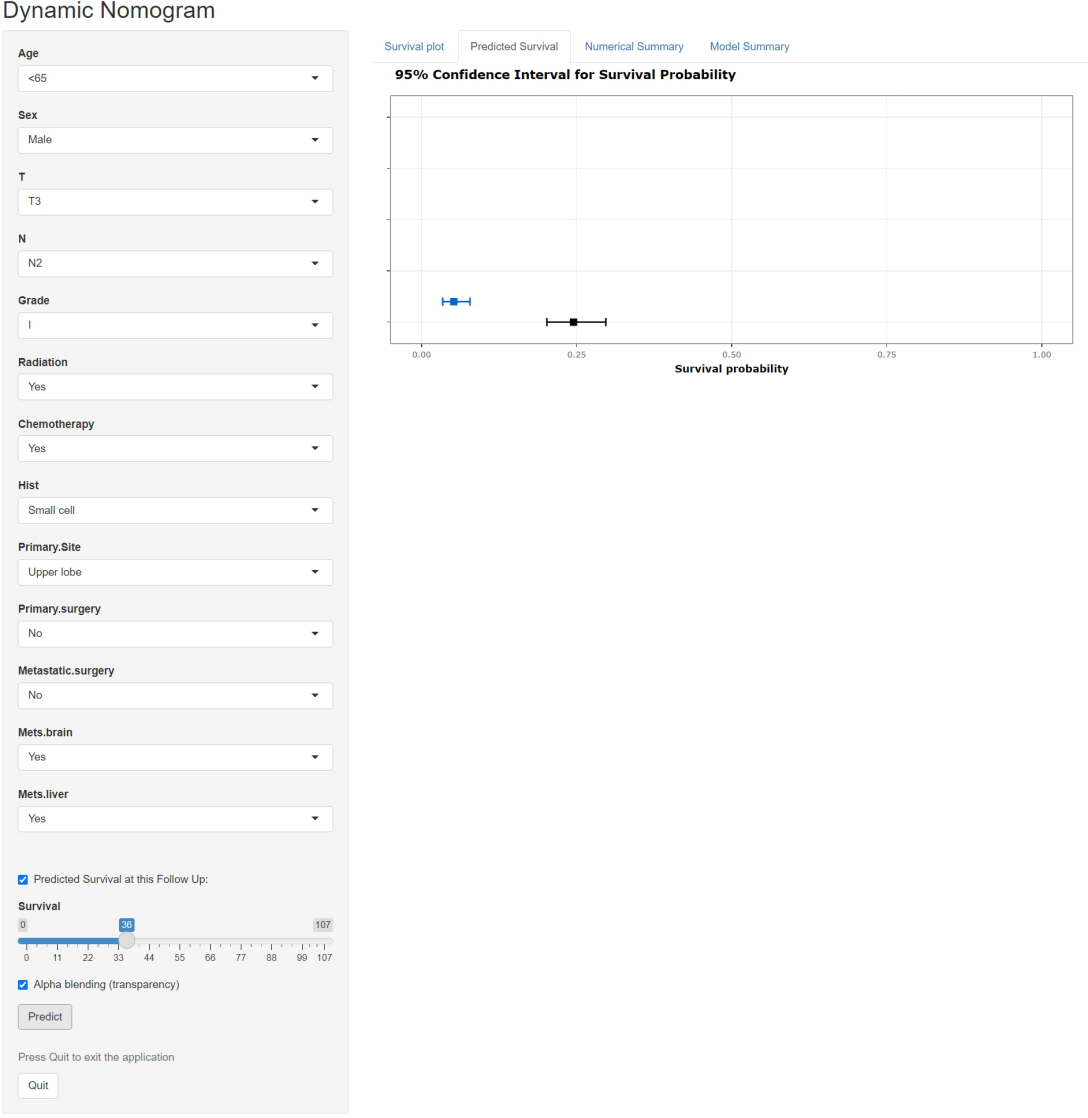
A web-based nomogram for predicting postoperative cancer-specific survival (CSS). The line segments of the graphical summary show the approximate range of overall survival rates.

A freely available, web-based calculator was deployed for predicting the postoperative OS and CSS of patients with LCBM (https://yiinmengchen.shinyapps.io/DynNom_os/ and https://yiinmengchen.shinyapps.io/DynNom_css/). With the use of the web-based nomogram, we can individually assess the OS and CSS of patients based on the input clinical factors. As an example of calculating OS, we included a 60-year-old male patient with LCBM with mets-brain and mets-liver. TNM stage was T3N2. Pathological grade was I. Histology was small cell. He received chemotherapy and radiation. As shown in **Figure 10**, the probability of OS for this patient was estimated to be 19.5% and 3.1% at 1 and 3 years, respectively. As shown in **Figure 11**, the probability of CSS for this patient was estimated to be 24.5% and 5.1% at 1 and 3 years, respectively.

## Discussion

Commonly, the symptoms associated with patients with LCBM are pain, occasional fractures, or interference with daily activities (20). BM accounts for approximately 350,000 deaths in the United States every year and nearly three times this number if patients in the European countries and Japan are also included (21). In the retrospective analysis of Swedish national inpatient data involving 21,169 patients with LC, by Riihimaki et al., BM conferred the worst prognosis compared with other frequent metastatic sites, which was one of the most frequent metastatic sites as well (22). In another study based on the nationwide Korean health insurance database, SREs most commonly occurred in patients with LC among the 1,849 patients with BM (23). The condition of patients with LCBM can rapidly progress to an advanced stage after initial diagnosis and display metastasis, which often renders the treatment difficult. Therefore, there is an urgent need for the development of more clinically applied risk predictors as well as novel tools for prediction of survival of patients with LCBM in the clinic. Based on the data extracted from the SEER database, we analyzed the survival of patients with LCBM. The prognostic factors associated with OS and CSS were also identified to accurately predict the prognosis of patients. As far as we know, this was the first multicenter population-based study that includes internal and external validation.

Our study found that some prognostic factors of LCBM were in accordance with previously published reports, including a male predominance, older age, and a propensity for high-grade tumors and more patients who were diagnosed with advanced stage III/IV disease (20, 24, 25). In addition to this, T, N, and M stages were correlated with shortened survival time. The effect of primary location of LC on prognosis after BM could not be defined, in accordance with our findings; the primary site was a factor associated with survival. We found that patients with main bronchial neoplasm had worse prognosis compared with other locations. Although there has been a previous study that confirmed that T3 centrally located early non–small cell LC (NSCLC) has a better survival than other types, more research studies obtained similar results with the present study (26, 27). This conclusion could be explained as the technical limitations of tumor resection involved in the main bronchus due to its anatomy. On the other hand, the main bronchus neoplasm had a high rate of lymph node metastasis, which was associated with worse outcomes (28).

As one of the most common metastatic sites, the bone is a unique microenvironment that appears to promote tumor growth. Our results revealed that liver and brain metastases were independent predictors of survival in LCBM. According to the theory of “seed and soil hypothesis,” metastatic cancer cells can dynamically interact with particular organ microenvironment and lead to different patterns of metastatic spread. Several studies found that the site of metastasis did not significantly influence patients’ survivals. However, our findings were supported by other researchers (20, 29). Tamura et al. reported a 1.55-fold increase in mortality in patients with liver metastasis compared with those with other metastasis (30). Most patients with LC with liver metastasis had multiple nodules morphologically and biliary tract obstruction may have been caused by LC metastatic to the lymph nodes in the porta hepatis or the hepatic parenchyma lesion. Patients would be jaundiced and would have a progressive divergence of hepatic synthetic and coagulation function. Meanwhile, the activation or metabolism of several cytotoxic drugs commonly used in various procedures for chemotherapy could be affected, in turn, leading to the limitation of chemotherapy’s administration. There were several cases reporting that patients with liver metastases could not receive conventional chemotherapy for liver dysfunction (31, 32). As one of the most common sites of metastasis in patients with LC, brain metastasis was regarded as one of the unfavorable prognostic factors in previous research studies. In one study, based on the SEER database, the cancer-specific case fatality was 91.01% after a median follow-up of 52 months in 5,974 patients with LC and with brain metastasis (33, 34). This study also revealed that patients with additional sites of metastasis (like BM) were related to worse survival. Thus, patients with LCBM combined with brain metastasis tend to exhibit poor prognosis. In the case of brain metastasis, the daily living activities of patients could be limited significantly and they could develop severe neurological symptoms, which may lead to reduced willingness of patients and doctors to pursue aggressive therapy. In addition, the use of chemotherapy for brain metastasis patients could be limited by poor efficacy and high toxicity. In the case of LCBM with brain metastasis, surgical treatment is not recommended because it has no significant impact on the long-term prognosis (32). In comparison, intracranial tumor biopsy is the gold standard for the diagnosis, which can determine not only the nature of intracranial lesions but also their source.

We found that radiotherapy, chemotherapy, and surgery of primary lesions were of prognostic significance in LCBM. Among them, chemotherapy contributed most significantly to prognosis in accordance with the nomogram. As is well known, chemotherapy remains the cornerstone of therapy in the management of advanced LC. First-line chemotherapy, maintenance chemotherapy, and second-line therapy were considered as regular therapeutic regimen to advanced NSCLC for many years (35, 36). Platinum-based doublets were used for the standard first-line chemotherapy, which could achieve symptoms remission and increased median survival by 1.5 months and the 1-year survival rate by 9%. Radiotherapy could relieve pain at the site of skeletal metastasis, reduce the incident of SRE, and could be used as an alternative treatment option for medically inoperable LC with high local control rates and with low toxicity (37). The technology of stereotactic body radiotherapy (SBRT) improved the accuracy and safety of radiotherapy for patients with LCBM, especially for those with spinal metastasis, which optimized radiation dose delivery to the BM while sparing the spinal cord.

We found that surgery of primary lesions was beneficial for prolonging both the OS and CSS of patients with LCBM. Although it is the standard treatment for patients with advanced LC, surgeons have performed curative resection in those who present with oligometastases. In the retrospective study by Takahashi et al., patients with NSCLC and synchronous isolated BMs achieved longer survival rates following primary lung tumor resection (38). However, other studies had different opinions. Patrini et al. suggested that there was no case in which BM was considered as an oligometastatic for the infaust prognosis (39). Considering quite the low number of patients who underwent surgery of the primary site, we speculated that they received surgery for oligometastases. The role of surgery in LCBM has not been effectively identified yet, especially for those with polymetastasis.

In addition to the previously mentioned treatments, bone-targeted pharmacological treatments including bisphosphonates and denosumab were widely used clinically to reduce pain and avoid SREs. In the last 30 years, bisphosphonate has been considered a key player in the therapy of BM from various cancers. Among bisphosphonates, zoledronic acid’s clinical effectiveness was validated in multiple studies (40). Compared with bisphosphonates, denosumab was found to be associated with delayed first and subsequent SREs and lower incidence of renal toxicity but higher incidence of hypocalcemia in several meta-analyses. However, we failed to consider the bone-targeted pharmacological treatments as the information in this regard was not provided by the SEER data center. In recent years, the application of next-generation sequencing technology has been widely used in the auxiliary diagnosis and target therapy of cancers (40). Epidermal growth factor receptor (EGFR) is the most widely used driving gene for the targeted treatment of LC and responds well to EGFR tyrosine kinase inhibitors (41). Previous studies have shown that microRNA, Dickkopf1, and insulin-like growth factor binding protein 3 are potential therapeutic targets for LCBM (40). Mukai et al. reported a high expression of mesenchymal-to-epithelial transition (MET) in both the primary metastasis and BM of patients with LC and suggested that drugs targeted at MET amplification, such as crizotinib and cabotinib, would have a certain effect on patients with LCBM (42). Recently, the study by Huang et al. found a high consistency of mutation patterns between primary LC lesions and matched BM, which indicated that the effective treatment of primary LC may also be suitable for matched LCBM, such as the EGFR-TKI treatment for LCBM with sensitive EGFR mutations (43). Unfortunately, the data of molecular alterations were not available in the SEER database, and our nomogram failed to include relevant factors.

Our study also has significant advantages. Compared with the previous studies, we identified the risk factors for BM in patients with LC and the prognostic factors of patients with LCBM. Meanwhile, we created a nomogram containing identified independent factors as a convenient and intuitive visual tool for prognostic prediction, which was verified by internal and external validation sets to guarantee the reliability of the results. As a retrospective cohort analysis with a large sample size, we point out that the validated results of current study can provide guidance to clinicians in daily routine practice and decision-making. However, some limitations are present. First, this was a retrospective study in which selection bias existed inevitably. Our study was limited by the data available in the SEER database. Second, in the process of patient screening, many failed to be enrolled to the SEER database for lack of detailed information like insurance and details on treatment. Missing data of these patients may mildly affect the accuracy of the research result. Third, the SEER database is based on the US population. The nomograms that we constructed may be limited by geographic constraints and may only be considered as a reference in the Chinese LCBM population. In the future, large multicenter studies should be performed in Chinese patients to develop a model to demonstrate its clinical validity for the Chinese population.

## Conclusion

In conclusion, the findings of this study based on a population level identified several factors that affect the OS and CSS of patients with LCBM, namely, age, sex, T stage, N stage, grade, histology, radiation therapy, chemotherapy, primary site, primary surgery, liver metastasis, and brain metastasis. We also found that metastatic surgery was beneficial for prolonging the CSS of patients with LCBM. Moreover, nomograms were developed to objectively predict 1-, 3, and 5-year OS and CSS of patients with this devastating disease. The result indicated that the nomogram had favorable discrimination, good consistency, and clinical benefits in patients with LCBM. For LCBM’s extremely poor prognosis, the development of the prediction models was important for patients and meant a lot to them. We point out that nomograms could help oncologists to make better clinical decisions and provide personalized treatment plans for patients.

## Data availability statement

The original contributions presented in the study are included in the article/supplementary material. Further inquiries can be directed to the corresponding authors.

## Author contributions

All authors were responsible for the study concept and design. MY, SG, XD, and RZ are co-first authors. JX and WM are co-response authors. All authors contributed to the article and approved the submitted version.
